# Metagenomics of Imported Multidrug-Resistant *Mycobacterium leprae*, Saudi Arabia, 2017

**DOI:** 10.3201/eid2603.190661

**Published:** 2020-03

**Authors:** Qingtian Guan, Talal S. Almutairi, Tahani Alhalouli, Arnab Pain, Faisal Alasmari

**Affiliations:** King Abdullah University of Science and Technology, Thuwal-Jeddah, Saudi Arabia (Q. Guan, A. Pain);; King Saud University Medical City, Riyadh, Saudi Arabia (T.S. Almutairi);; King Fahad Medical City, Riyadh (T.S. Almutairi, T. Alhalouli, F. Alasmari)

**Keywords:** *Mycobacterium leprae*, metagenomics, drug resistance, imported infectious diseases, tuberculosis and other mycobacteria, *rpoB*, *rrs*, Philippines, Saudi Arabia, single-nucleotide polymorphisms, indels, antimicrobial resistance, leprosy, bacteria, biopsy, 3K1

## Abstract

Using shotgun metagenomics, we identified an imported case of multidrug-resistant *Mycobacterium leprae* in a Filipino resident of Saudi Arabia in 2017. We determined the phylogenomic lineage (3K1) and identified mutations in *rpoB* and *rrs* corresponding to the multidrug-resistance phenotype clinically observed. Metagenomics sequencing can be used to identify multidrug-resistant *M. leprae*.

Leprosy is a chronic dermatologic and neurologic disease caused by the infectious agent *Mycobacterium leprae* and can lead to severe disabilities; >200,000 new cases are reported annually worldwide, according to the World Health Organization. A total of 242 leprosy cases were reported in Saudi Arabia during 2003–2012; however, little is known about the subtypes and prevalence of drug resistance among these *M. leprae* cases (*1*).

In May 2017, a 30-year-old woman from the Philippines sought treatment at the dermatology clinic of King Fahad Medical City (KFMC) Hospital in Riyadh, Saudi Arabia, for painful systemic skin nodules and joint pain without joint swelling. She had no medical history of leprosy. The initial clinical diagnosis of this patient was inconclusive, but her initial signs and symptoms were suggestive of a connective tissue disease such as systemic lupus erythematosus, and initial clinical improvement was recorded after a short course of empiric steroids and hydroxychloroquine treatment. Other suspected diagnoses included lepromatous leprosy with type 2 erythema nodosum leprosum reaction or other nontuberculosis mycobacterial infection.

We performed a punch skin biopsy of the extensor surface of the forearm and performed Ziehl-Neelsen staining; we observed a florid histiocytic proliferation containing numerous *Mycobacterium* bacilli without an obvious granuloma (Appendix Figure 1). We referred the patient to the infectious disease clinic, which performed QuantiFERON-TB Gold (QIAGEN, https://www.quantiferon.com) and took a biopsy for bacterial and fungal culture, and all test results were negative. Mycobacterial culture showed >9 acid-fast bacilli/high-power field on smears, and no growth was observed on Lowenstein-Jensen slants after 8 weeks of incubation.

Her treatment started with a daily regimen of clofazimine (50 mg), dapsone (100 mg), and rifampin (600 mg). Treatment with moxifloxacin (400 mg/d) and macrolides was briefly added (clarithromycin and azithromycin were both stopped because of gastrointestinal side effects) in case of possible nontuberculosis mycobacterial infection. The patient had multiple relapses during 12 months of follow-up and became steroid dependent (i.e., her skin lesions reappeared shortly after steroid treatment ended).

Because initial test reports were inconclusive and the etiologic agent was unconfirmed, we attempted to confirm the etiology by subjecting the patient’s skin biopsy sample to metagenomic sequencing; a DNA sequencing protocol without target DNA–enrichment steps (*2*) was needed to unambiguously identify the etiologic agent. From the metagenomics datasets, we reconstructed the near-complete genome of the *M. leprae* species (which we named KFMC-1) at 99.2% completeness when compared with *M. leprae* TN, a strain commonly used for reference (*3*). We assembled the 3.24-Mb genome of *M. leprae* KFMC-1 in 19 DNA segments, and average coverage was 20.02× (Appendix Table 1, Figure 2, panel A). A single-nucleotide polymorphism comparison of *M. leprae* KFMC-1 with a globally representative set of *M. leprae* revealed KFMC-1 was most closely related to 3K1 Ryukyu-2 (Appendix Figure 2, panel B), which was originally isolated in Japan (*4*). 

We identified 158 polymorphic sites in the genome (Appendix Table 2), which corresponded to 136 single-nucleotide polymorphisms and 22 insertion/deletions. In total, 53 of the 158 changes were new, and 63 appeared within gene-coding regions, a couple of which helped us predict the multidrug-resistance profile. We identified a G→T nucleotide change, which leads to a nonsynonymous change (Q438H) in the *rpoB* gene (Appendix Figure 2, panel C). This substitution results in rifampin resistance (*5*), matching our clinical records. The C1414A mutation in the *rrs* locus is predicted to confer capreomycin resistance, as observed previously in *M. tuberculosis* (*6*). 

After we confirmed the clinical diagnosis as an *M. leprae* infection, we halted moxifloxacin treatment and kept the patient on 3 standard antimicrobial drugs (clofazimine, dapsone, and rifampin). Afterward, the patient left Saudi Arabia and continued her antimicrobial drug course in her country of origin.

The predominant genotypes of *M. leprae* strains in the Middle East are subtypes 2 and 3 (*7*). Most 3K cases are found in countries of East Asia, such as China (*8*), Japan (*9*), Korea (*2*), and the Philippines (*8*). In addition, >37% of the leprosy cases in Saudi Arabia occur in persons from other countries (*1*). Our results suggest that this case of leprosy was imported from the patient’s country of origin. Saudi Arabia hosts a massive number of expatriates from all over the world, including persons from *M. leprae*–endemic countries, and also hosts one of the largest recurring religious gatherings in the world. Therefore, genomics-guided infection control efforts are needed to monitor the potential importation and prevent the spread of *M. leprae* infections in the region.

Appendix More information about metagenomics of imported multidrug-resistant *Mycobacterium leprae*, Saudi Arabia, 2017.
