# Supplementary material for: Metagenomics of Imported Multidrug-Resistant Mycobacterium leprae, Saudi Arabia, 2017
Source: Emerg Infect Dis. 2020 Mar;26(3):615–7. doi: 10.3201/eid2603.190661 (PMC7045828; doi:10.3201/eid2603.190661)
Supplement: Appendix — More information about metagenomics of imported multidrug-resistant Mycobacterium leprae, Saudi Arabia, 2017. [file 19-0661-Techapp-s1.pdf]

Article DOI: https://doi.org/10.3201/eid2603.190661

Metagenomics of Imported Multidrug-Resistant *Mycobacterium leprae*, Saudi Arabia, 2017

Appendix

Materials and Methods

The research protocol was approved by the Institutional Review Board of King Fahad Medical City (Riyadh, Saudi Arabia; #16–345) and Institutional Biosafety and Bioethics Committee of King Abdullah University of Science and Technology (Jeddah, Saudi Arabia; #18IBEC23).

The DNA from the patient sample was extracted using the ZymoBIOMICS DNA/RNA Miniprep Kit (Zymo Research, Freiburg, Germany). NuGEN Ovation Ultralow Library System V2 (NuGen, Manchester, UK) was used for library preparation. A total of 3.51 billion 150 bp pair-end Illumina reads were obtained from Illumina Hiseq4000 instrument. We performed the reference-guided assembly using *M. leprae* TN strain as the reference. Single-nucleotide polymorphisms (SNPs) were called for two iterations and filtered with Genome Analysis Toolkit (*1*) (GATK) which had a read depth of 3 or higher; an alignment quality score (MQ) ≥40; SNPs

subtyping of *M. leprae* KFMC-1 was done based on SNP subtypes (A–P) defined by surveying the informative SNPs described by Marc Monot et al. in 2009 (*2*). Phylogenies were generated by aligning all SNPs from representative genomes from various linages of leprosy genomes (*3*) using maximum likelihood.

The SNP in the *rpoB* gene was examined by nested PCR of 10 ng of genomic DNA with the method described (*4*) and the C1414A variant in *rrs* was verified with semi-nested PCR with 98°C for 2 min followed by 35 cycles of denaturation (98°C for 10 sec), primer annealing (62°C, 30 sec for first-round PCR, forward 5′-CGCGTTGTTCGTGAAATCT-3, reverse 5′-ATGCTCGCAACCACTATCCA-3; or 60°C 30 sec for second-round PCR, 1492R GGCTACCTTGTTACGACTT) and extension (72°C, 30 sec), and final extension at 72°C for 2 min. A PCR premix (Q5 Hot Start High-Fidelity 2X Master Mix, New England Biolabs).

Appendix References

1. Alkan C, Coe BP, Eichler EE. Genome structural variation discovery and genotyping. Nat Rev Genet. 2011;12:363–76. PubMed http://dx.doi.org/10.1038/nrg2958

2. Monot M, Honoré N, Garnier T, Zidane N, Sherafi D, Paniz-Mondolfi A, et al. Comparative genomic and phylogeographic analysis of *Mycobacterium leprae*. Nat Genet. 2009;41:1282–9. PubMed https://doi.org/10.1038/ng.477

3. Benjak A, Avanzi C, Singh P, Loiseau C, Girma S, Busso P, et al. Phylogenomics and antimicrobial resistance of the leprosy bacillus *Mycobacterium leprae*. Nat Commun. 2018;9:352. PubMed https://doi.org/10.1038/s41467-017-02576-z

4. Kai M, Nguyen Phuc NH, Nguyen HA, Pham THBD, Nguyen KH, Miyamoto Y, et al. Analysis of drug-resistant strains of *Mycobacterium leprae* in an endemic area of Vietnam. Clin Infect Dis. 2011;52:e127–32. PubMed https://doi.org/10.1093/cid/ciq217

Appendix Table 1. Comparison of *M. leprae* KFMC-1 and *M. leprae* TN assemblies*

Category	*M. leprae* KFMC	*M. leprae* TN
Sequence size	3243015	3268203
Number of contigs	19	1
GC content (%)	57.8	57.8
contigs (> = 50000 bp)	14	1
Median sequence size	94825	3268203
Mean sequence size	141002.1	3268203
Longest contig size	497079	3268203
N50 value	318796	NA
L50 value	5	1

*KFMC, King Fahad Medical City; NA, not applicable.

Appendix Table 2. SNPs/INDEL present in *M. leprae* KFMC-1*

POS	Type	REF (TN)	ALT (KFMC)	Novelty	Annotation	ML code
73	SNP	T	G	No	missense_variant	ML0001
12484	Indel	A	AACCACAGCTAGAC	No	intergenic_region	MLP000002-ML0008c
14226	Indel	C	CATAT	Yes	intergenic_region	ML0009-ML0010c
15439	SNP	G	A	No	pseudogene	ML0010c
17425	SNP	G	A	Yes	intergenic_region	ML0013c-ML0014
26545	SNP	G	A	No	synonymous_variant	ML0020c
40852	SNP	G	T	Yes	pseudogene	ML0034
52851	SNP	T	C	No	missense_variant	ML0042
57633	SNP	T	G	No	pseudogene	ML0046c
61425	SNP	A	G	No	missense_variant	ML0049c
62545	SNP	G	T	No	missense_variant	ML0051c
73073	Indel	CGATCAA, GCCAGGA, ATCAAGT, TGATCAA, GCCAGGA, ATCAAGTT	C	Yes	pseudogene	ML0058c
77864	SNP	T	C	No	synonymous_variant	ML0061
86658	SNP	G	A	Yes	intergenic_region	ML0064c-ML0065
100574	SNP	A	G	No	pseudogene	ML0080c
132150	SNP	G	A	Yes	synonymous_variant	ML0102
157960	SNP	C	T	No	intergenic_region	ML0116c-ML0117
160627	SNP	G	T	Yes	synonymous_variant	ML0119c
175636	SNP	C	T	Yes	missense_variant	ML0131
286105	SNP	C	T	Yes	synonymous_variant	ML0214
313361	SNP	A	G	No	missense_variant	ML0238c
328634	SNP	G	C	No	missense_variant	ML0252
330125	SNP	G	A	No	missense_variant	ML0252

POS	Type	REF (TN)	ALT (KFMC)	Novelty	Annotation	ML code
365435	SNP	G	A	No	missense_variant	ML0283
383599	SNP	C	G	No	pseudogene	ML0301c
441420	Indel	CAT	C	Yes	intergenic_region	ML0349c-ML0350c
459887	SNP	C	T	No	pseudogene	ML0368
481476	SNP	A	G	No	synonymous_variant	ML0387
494674	SNP	T	G	No	missense_variant	ML0397c
504437	SNP	G	C	Yes	intergenic_region	ML0405-ML0406
508481	SNP	T	C	No	missense_variant	ML0410
509325	SNP	C	G	No	missense_variant	ML0411
517971	SNP	C	T	Yes	intergenic_region	ML0841-ML0842
528451	SNP	C	T	No	pseudogene	ML0428
533403	SNP	A	G	No	pseudogene	ML0433
561823	Indel	G	GGT	Yes	pseudogene	ML0463c
686240	SNP	A	C	No	pseudogene	ML0567
694090	SNP	T	C	No	missense_variant	ML0569c
711197	SNP	T	C	No	pseudogene	ML0585c
714396	SNP	C	T	No	synonymous_variant	ML0589c
736703	SNP	G	T	Yes	missense_variant	ML0605
790218	SNP	C	T	Yes	pseudogene	ML0652
831215	SNP	G	T	No	pseudogene	ML0693
832152	SNP	T	C	No	pseudogene	ML0694c
890453	SNP	A	G	No	missense_variant	ML0747c
904824	SNP	G	C	No	synonymous_variant	ML0763
938372	SNP	C	T	No	pseudogene	ML0794c
944191	Indel	C	CA	Yes	pseudogene	ML0797c
958228	Indel	A	AC	Yes	pseudogene	ML0809
972005	SNP	T	G	No	pseudogene	ML0821
1000186	SNP	C	T	Yes	intergenic_region	ML0841-ML0842
1041037	SNP	C	T	Yes	synonymous_variant	ML0876

POS	Type	REF (TN)	ALT (KFMC)	Novelty	Annotation	ML code
1066586	SNP	T	C	Yes	pseudogene	ML0900c
1076949	SNP	G	C	No	missense_variant	ML0909
1087397	SNP	T	C	No	synonymous_variant	ML0917
1104232	SNP	C	G	No	pseudogene	ML0934
1133492	SNP	T	G	No	intergenic_region	ML0964-ML0965c
1133721	Indel	C	CG	Yes	intergenic_region	ML0964-ML0965C
1143423	SNP	T	C	No	pseudogene	ML0975c
1144840	SNP	T	G	Yes	synonymous_variant	ML0977
1155582	SNP	T	G	No	synonymous_variant	ML0988
1227051	SNP	G	T	No	intergenic_region	ML1061-ML1062
1257185	SNP	T	C	No	intergenic_region	ML1092c-ML1093
1265267	SNP	T	G	No	pseudogene	ML1097
1295192	SNP	A	G	No	missense_variant	ML1119
1324009	SNP	C	G	No	missense_variant	ML1132
1339813	SNP	T	C	No	synonymous_variant	ML1150
1342557	SNP	C	A	Yes	non_coding_transcript_exon_variant	MLP000016
1348426	SNP	T	C	No	intergenic_region	ML1152c-ML1153
1351149	SNP	C	G	No	intergenic_region	ML1154c-ML1155
1529088	SNP	A	G	No	pseudogene	ML1284c
1532258	SNP	G	A	No	missense_variant	ML1286
1533315	Indel	C	CG	Yes	pseudogene	ML1287
1587912	SNP	G	T	Yes	missense_variant	ML1334
1605956	SNP	G	A	No	intergenic_region	ML1345-ML1346
1607562	SNP	A	G	Yes	missense_variant	ML1346
1614069	SNP	T	C	No	pseudogene	ML1353c
1625045	SNP	T	G	No	synonymous_variant	ML1363
1640789	SNP	A	G	Yes	pseudogene	ML1376
1642875	SNP	G	T	No	pseudogene	ML1378
1643162	SNP	T	C	No	pseudogene	ML1378

POS	Type	REF (TN)	ALT (KFMC)	Novelty	Annotation	ML code
1677493	SNP	G	C	No	synonymous_variant	ML1397
1700105	SNP	T	C	Yes	missense_variant	ML1417
1701590	SNP	G	C	No	intergenic_region	ML1418c-ML1419c
1725797	SNP	C	G	No	pseudogene	ML1436c
1741830	Indel	G	GCAACAACGTC	Yes	pseudogene	ML1449c
1813428	Indel	A	AGT	No	pseudogene	ML1502c
1839270	SNP	A	G	No	pseudogene	ML1524c
1841279	Indel	C	CG	Yes	pseudogene	ML1527c
1843283	SNP	C	A	No	pseudogene	ML1528
1868993	SNP	G	T	No	pseudogene	ML1545
1876289	SNP	G	A	Yes	pseudogene	ML1552
1926696	SNP	T	C	No	intergenic_region	ML1600c-ML1601c
1955004	SNP	C	A	Yes	synonymous_variant	ML1629
1971193	SNP	G	A	No	pseudogene	ML1636
2011747	SNP	T	G	No	pseudogene	ML1668c
2011783	SNP	G	A	Yes	pseudogene	ML1668c
2030803	SNP	G	A	No	missense_variant	ML1685c
2040883	SNP	G	A	No	pseudogene	ML1693
2043287	SNP	A	G	No	synonymous_variant	ML1694c
2066936	SNP	G	T	Yes	missense_variant	ML1713
2100523	SNP	C	G	No	missense_variant	ML1740c
2104127	SNP	T	C	No	intergenic_region	ML1743c-ML1744c
2142011	Indel	CT	C	No	pseudogene	ML1767c
2148809	SNP	G	A	No	pseudogene	ML1773c
2155013	SNP	T	G	No	pseudogene	ML1778c
2174865	SNP	G	C	No	intergenic_region	ML1795-ML1796
2205779	Indel	TA	T	No	pseudogene	ML1822
2211034	Indel	TAC	T	Yes	intergenic_region	ML1825c-ML1826c
2235048	SNP	C	T	No	pseudogene	ML1851c

POS	Type	REF (TN)	ALT (KFMC)	Novelty	Annotation	ML code
2263547	SNP	G	A	No	intergenic_region	ML1883-ML1884
2275492	SNP	C	A	Yes	missense_variant	ML1891c
2278551	SNP	C	A	No	pseudogene	ML1893c
2312059	SNP	C	G	No	start_lost	ML1926c
2317249	SNP	C	T	Yes	pseudogene	ML1933
2344787	SNP	G	A	No	synonymous_variant	ML1957c
2441339	SNP	C	T	No	missense_variant	ML2053c
2459766	SNP	A	G	No	missense_variant	ML2069
2468130	SNP	C	A	No	missense_variant	ML2075c
2514637	SNP	C	T	Yes	intergenic_region	ML2114c-ML2115c
2515916	SNP	G	T	Yes	pseudogene	ML2115c
2526543	SNP	G	T	Yes	pseudogene	ML2125c
2547925	SNP	C	G	Yes	pseudogene	ML2145
2553176	SNP	T	G	No	pseudogene	ML2149
2567248	Indel	AGTG	A	No	intergenic	ML2159c-ML2160
2631245	SNP	G	A	Yes	synonymous_variant	ML2213c
2651703	SNP	C	A	Yes	intergenic_region	ML2233-ML2234
2691666	SNP	G	A	Yes	pseudogene	ML2267
2706236	SNP	T	G	No	pseudogene	ML2281c
2711942	SNP	T	G	No	pseudogene	ML2287c
2747358	SNP	T	C	Yes	missense_variant	ML2320c
2751783	SNP	A	G	No	synonymous_variant	ML2322c
2757405	Indel	GC	G	No	pseudogene	ML2325c
2804726	SNP	C	A	No	synonymous_variant	ML2354c
2818521	SNP	T	C	No	synonymous_variant	ML2357c
2835913	Indel	CGTGT	C	Yes	pseudogene	ML2367
2844969	Indel	CAT	C	Yes	intergenic_region	ML2375c-ML2376c
2887094	Indel	C	CATA	Yes	intergenic_region	ML2415-ML2416c
2935685	SNP	A	C	No	pseudogene	ML2462c

POS	Type	REF (TN)	ALT (KFMC)	Novelty	Annotation	ML code
2964999	SNP	T	C	No	synonymous_variant	ML2490c
2981212	SNP	G	A	No	synonymous_variant	ML2501
3016175	SNP	T	C	No	synonymous_variant	ML2534c
3057114	SNP	G	C	No	intergenic_region	ML2563-ML2564c
3063817	SNP	C	A	Yes	missense_variant	ML2568c
3076050	SNP	G	C	No	intergenic_region	ML2574c-ML2575c
3102778	SNP	A	C	No	missense_variant	ML2597
3132639	SNP	G	A	No	synonymous_variant	ML2622c
3152586	SNP	C	T	No	synonymous_variant	ML2634c
3175296	SNP	A	C	No	intergenic_region	ML2652-ML2653
3202695	SNP	A	G	No	intergenic_region	ML2670c-ML2671
3221210	SNP	G	A	Yes	pseudogene	ML2676c
3221615	Indel	AATAT	A	Yes	intergenic_region	ML2676c-ML2677
3236317	SNP	G	A	No	missense_variant	ML2687c
3243731	SNP	A	G	No	pseudogene	ML2694
3254050	SNP	C	T	Yes	synonymous_variant	ML2700
3256572	SNP	C	T	No	synonymous_variant	ML2700
3257047	Indel	GCCCA	G	Yes	pseudogene	ML2701
3268175	SNP	G	T	No	intergenic_region	ML2713c-ML0001

*ALT, alternative; KFMC, King Fahad Medical City; POS, position; REF, reference; SNP, single-nucleotide polymorphism.

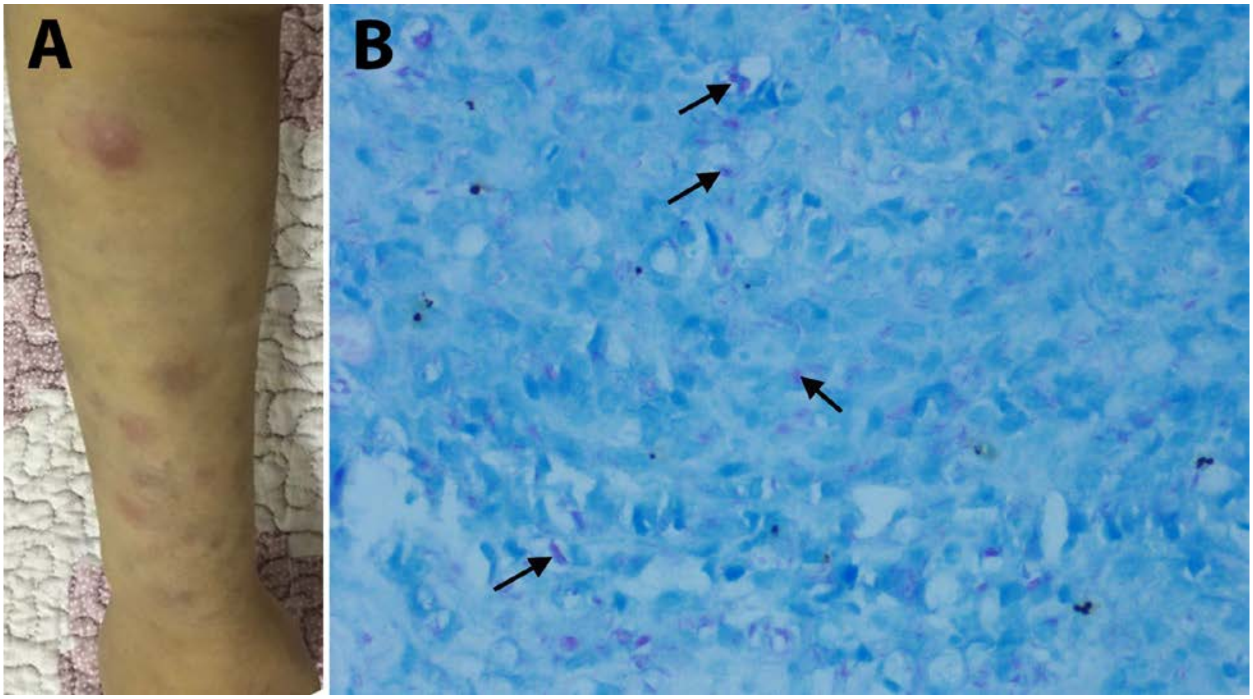

Appendix Figure 1. A) Photographs of forearm skin lesions. B) Histopathology of the skin biopsy using Ziehl-Neelsen staining (100X). Arrows showing *M. leprae* bacilli.

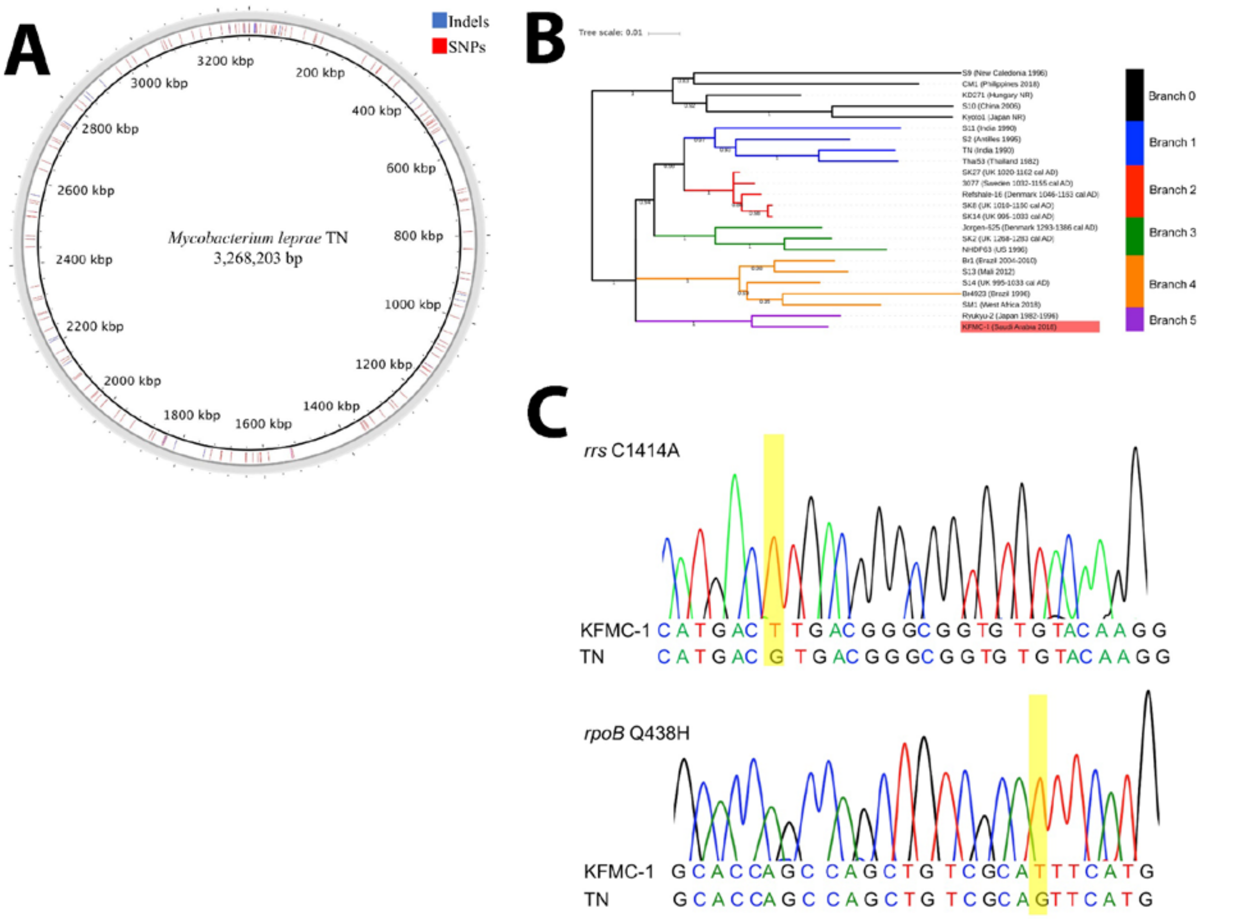

Appendix Figure 2. A) *M. leprae* KFMC-1 genome comparison against the *M. leprae* TN as the reference genome. BLASTn matches above 90% nucleotide identity are colored in gray. The vertical bars represent the polymorphic sites in *M. leprae* KFMC-1 when compared to the *M. leprae* TN strain. The SNP positions in the genome are shown in red while the nucleotide insertions and deletions (Indels) shown in blue; The gray outer circle shows *M. leprae* KFMC-1 shared identity (according to BLASTn) with *M. leprae* TN genome as the reference. B) Phylogenetic lineages of *M. leprae* KFMC-1 with the representatives from *M. leprae* isolates based on SNP sites using a maximum likelihood approach using the Tamura-Nei model. Bootstrap percentages from 1,000 replicates are shown next to the branches. The scale indicates the

number of substitutions per site. C) Chromatograms of *M. leprae* KFMC-1 showing the Q438H mutation in *rpoB* and C1414A mutation in *rrs*. KFMC, King Fahad Medical City; SNP, single-nucleotide polymorphism.
